# Prevalence of loss-of-function alleles does not correlate with lifetime fecundity and other life-history traits in metazoans

**DOI:** 10.1186/s13062-018-0206-9

**Published:** 2018-03-02

**Authors:** Aleksandra V. Bezmenova, Georgii A. Bazykin, Alexey S. Kondrashov

**Affiliations:** 1 Center for Data-Intensive Biomedicine and Biotechnology, Skolkovo Institute of Science and Technology, Moscow, 143026 Russia; 2 Laboratory of Evolutionary Genomics, A.N. Belozersky Institute of Physico-Chemical Biology of Lomonosov Moscow State Universit, Moscow, 119992 Russia; 3 Sector for Molecular Evolution, Kharkevich Institute of Information Transmission Problems of the Russian Academy of Sciences, Moscow, 127051 Russia; 4 Department of Ecology and Evolutionary Biology, University of Michigan, Ann Arbor, MI 48109 USA

**Keywords:** Selection strength, Negative selection, Loss-of-function, Fecundity, Life-history traits

## Abstract

**Background:**

Natural selection is possible only because all species produce more offsprings than what is needed to maintain the population. Still, the lifetime number of offspring varies widely across species. One may expect natural selection to be stronger in high-fecundity species. Alternatively, natural selection could be stronger in species where a female invests more into an individual offspring. This issue needed to be addressed empirically.

**Results:**

We analyzed the prevalence of loss-of-function alleles in 35 metazoan species and have found that the strength of negative selection does not correlate with lifetime fecundity or other life-history traits.

**Conclusions:**

Higher random mortality in high-fecundity species may negate the effect of increased opportunity for selection. Perhaps, invariance of the strength of negative selection across a wide variety of species emerges because natural selection optimized the life history in each of them, leading to the strongest possible competition.

**Reviewers:**

This article was reviewed by Nicolas Galtier and I. King Jordan.

**Electronic supplementary material:**

The online version of this article (10.1186/s13062-018-0206-9) contains supplementary material, which is available to authorized users.

## Background

In the long run, the size of every population that does not go extinct remains approximately constant. Thus, in the course of many generations the geometric mean number of daughters of a female surviving to reproduce must always be one. However, lifetime fecundity of all species is way above this minimum. “There is no exception to the rule that every organic being naturally increases at so high a rate, that if not destroyed, the earth would soon be covered by the progeny of a single pair” [1]. In some species, such as elephants and bears, the maximal lifetime fecundity is only ~ 10, while many others can produce millions of offspring. Of course, to ensure a constant long-term population size, pre-reproductive mortality in a species must be proportional to its average lifetime fecundity.

Production of excessive offspring is a *sine qua non* of natural selection. Indeed, in a species where the maximal lifetime number of daughters is one, any selection would lead to extinction, which could be avoided only if every female produces exactly one daughter. Quantitatively, selection always induces some positive genetic load $L=\frac{w_{max}-W}{w_{max}}$, where *w*_*max*_ is the maximal possible fitness and *W* is the mean population fitness [2], and, in order for the population size to remain stable, the expected lifetime number of successful daughters of females with the highest fitness must be $\frac{1}{1-L}$. The actual maximal number of daughters of a female must be even larger, due to their pre-reproductive mortality and to random variation of reproduction success among females with the same genotype.

Thus, there is less limitation on the strength of selection in high-fecundity species, which can sacrifice a larger proportion of offspring without going extinct, compared to low-fecundity ones. Therefore, one may expect selection to be stronger in the former. However, this is not necessarily the case, because in high-fecundity species parental investment in an offspring is necessarily low, so that their random mortality must be higher. Thus, while in low-fecundity species mortality of offspring may be mostly due to imperfection of their genotypes and thus lead to selection, in high-fecundity species the bulk of mortality may be irrelevant to selection.

Clearly, the relationship between the maximal fecundity, as well as other life-history traits, of a species and the strength of selection in it needs to be established empirically. With this goal in mind, we compared strengths of negative selection against loss-of-function (LoF) alleles of orthologous genes in 35 metazoan species.

## Methods

We used a large set of transcriptomes published by [3], which consists of sequences of 374 individuals from 76 metazoan species representing 6 phyla (Cnidaria, Annelida, Mollusca, Arthropoda, Echinodermata, and Chordata). This dataset also contains information about a number of life-history traits (LHT), such as adult size, body mass, longevity, fecundity, and propagule size. We also collected information about genome sizes of these species, when available [4].

Raw reads were downloaded from the SRA database; SRA accession numbers are listed in Additional file 1: Table S1. They were trimmed of low-quality positions and sequencing adapters with Trimmomatic software [5]. Individuals that failed to pass quality control by fastQC [6] after trimming were excluded. Reads from all individuals that belong to a species were pooled together and de novo assembled into contigs using Trinity [7]. Trinity may produce several isoforms of a gene. To exclude minor isoforms, reads were mapped to the assemblies using Bowtie2 [8] and FPKM values were calculated using RSEM program [9]. For each gene, we chose an isoform with the highest FPKM value. Open reading frames (ORFs) were predicted using Transdecoder program (with minimum protein length set to 100 amino acids). If more than one ORF were predicted in a contig, the longest ORF was used.

We focused on those genes, hereinafter referred to as core gene, that are present in the list of essential genes of metazoans [10]. A subset of core genes, further referred to as hard-core genes, was obtained by excluding those genes that harbor at least one homozygous LoF allele in at least one species. Information about species assemblies (number of contigs, N50, mean coverage, alignment rate) and annotated coding sequences (numbers of predicted ORFs and of core genes) is presented in Additional file 2: Table S2. Species with reads alignment rates below 70%, numbers of predicted ORFs below 5000, or the numbers of predicted core genes below 100 were excluded.

For each individual separately, reads were mapped to the reference assembly of the species using Bowtie2. Individuals with the mean coverage below 10X, reads alignment rate below 80%, number of (at least partially) covered ORFs below 5000 or the number of (at least partially) covered core genes below 100 were excluded. SNPs and small indels were called using Samtools mpileup [11] and annotated with Annovar [12]. Only positions with depth more than 5× and mapping and variant calling quality over 20 were considered valid. Stopgain and stoploss substitutions and frameshift indels were assumed to be loss-of-function variants (LoFs).

For each individual, we calculated the proportion of genes that carry at least one LoF variant (LoF alleles) among all predicted ORFs and among core genes. Not all ORFs annotated in reference transcriptomes were sequenced in each individual. The proportion of LoF alleles in an individual was calculated as $p=\frac{N_{LoFHet}+2{N}_{LoFHom}}{2N}$, were *N* is the number of ORFs fully sequenced for the individual; *N*_*LoFHet*_ is the number of heterozygous LoF alleles and *N*_*LoFHom*_ is the number of homozygous LoF alleles. This proportion was calculated for all predicted ORFs and for the subsets of core and hard-core genes.

After applying the filters described above, and excluding 4 species of Hymenoptera whose males are haploid, we ended up with the data set consisting of 35 species, represented by between 1 and 9 individuals, with median 2 (Additional file 2: Table S2). LHTs of these species as well as genome sizes and synonymous nucleotide diversities (*πs*) obtained from [3] are shown in Additional file 3: Table S3. Spearman correlation coefficients for proportions of LoF alleles vs. different LHTs were calculated in R; *p*-values were corrected for multiple testing using Benjamini and Hochberg procedure.

## Results

We recorded the numbers of LoF alleles in genotypes of between 1 and 9 individuals from 35 metazoan species. The proportions of LoF alleles among all predicted genes, core genes, and hard-core genes are shown in Additional file 4: Table S4 (for each individual) and in Additional file 5: Table S5 (mean values for each species). These proportions vary from 0.34 to 5.33% for all genes, from 0 to 5.36% for core genes, and from 0% to 1.85% for hard-core genes, with the means being 2.21, 1.08 and 0.22%, respectively. The mean proportion of alleles that carry nonsense substitutions among all genes in all species was 0.44%, while the mean proportion of alleles that carry frameshift indels was 1.66% (Table 1).

**Table 1:** Mean proportions of LoF alleles of all genes in a species

	Min	Max	Mean
All LoF alleles	0.34%	5.33%	2.21%
Nonsense	0.07%	1.03%	0.44%
Frameshift	0.18%	4.27%	1.66%
Stoploss	0.00%	0.66%	0.12%

**Fig. 1:**
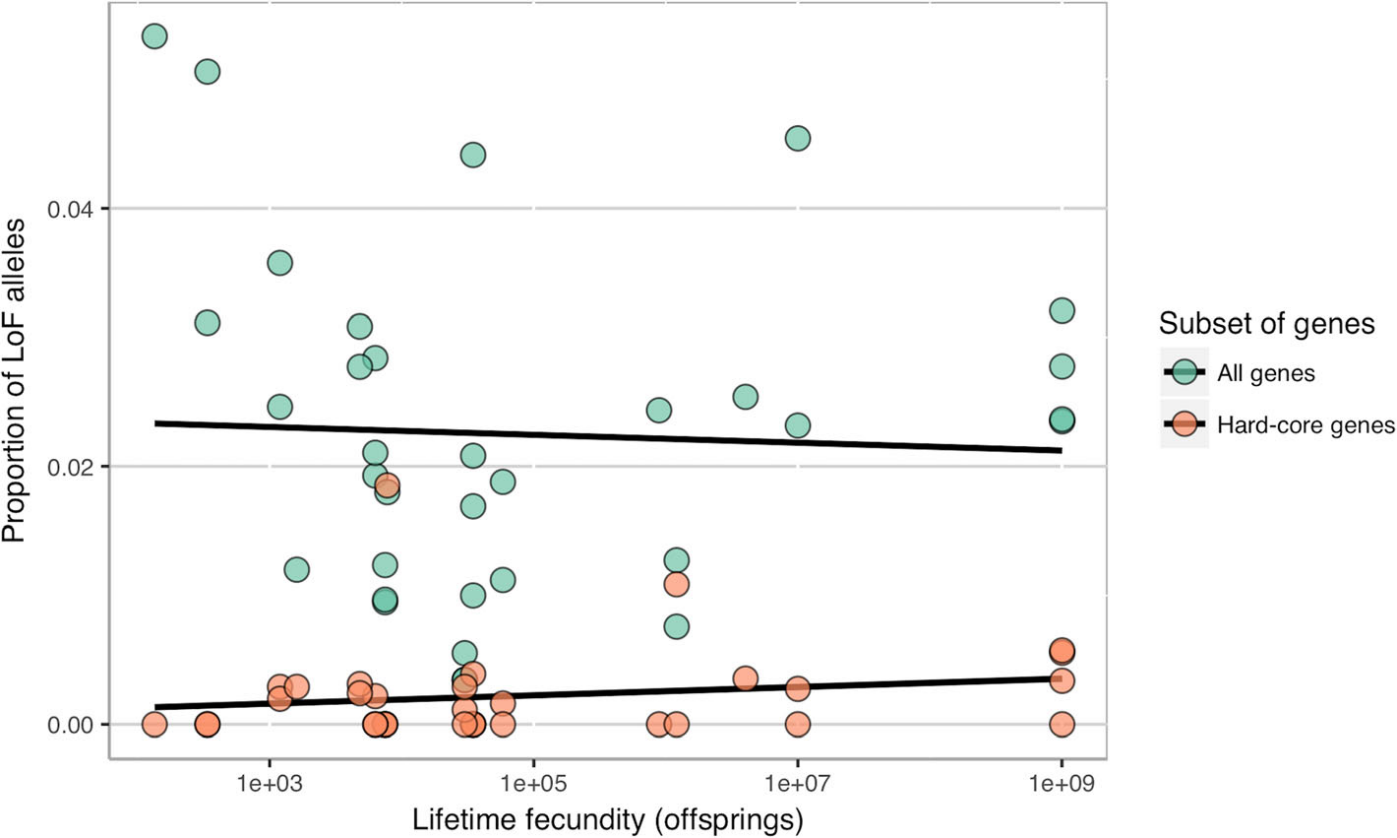
The mean proportions of LoF alleles against lifetime fecundity in all (green) and in hard-core (orange) genes for each species (Spearman’s correlation coefficients are −0.14 and 0.22, respectively; *p*-values are 0.41 and 0.21)

**Fig. 2:**
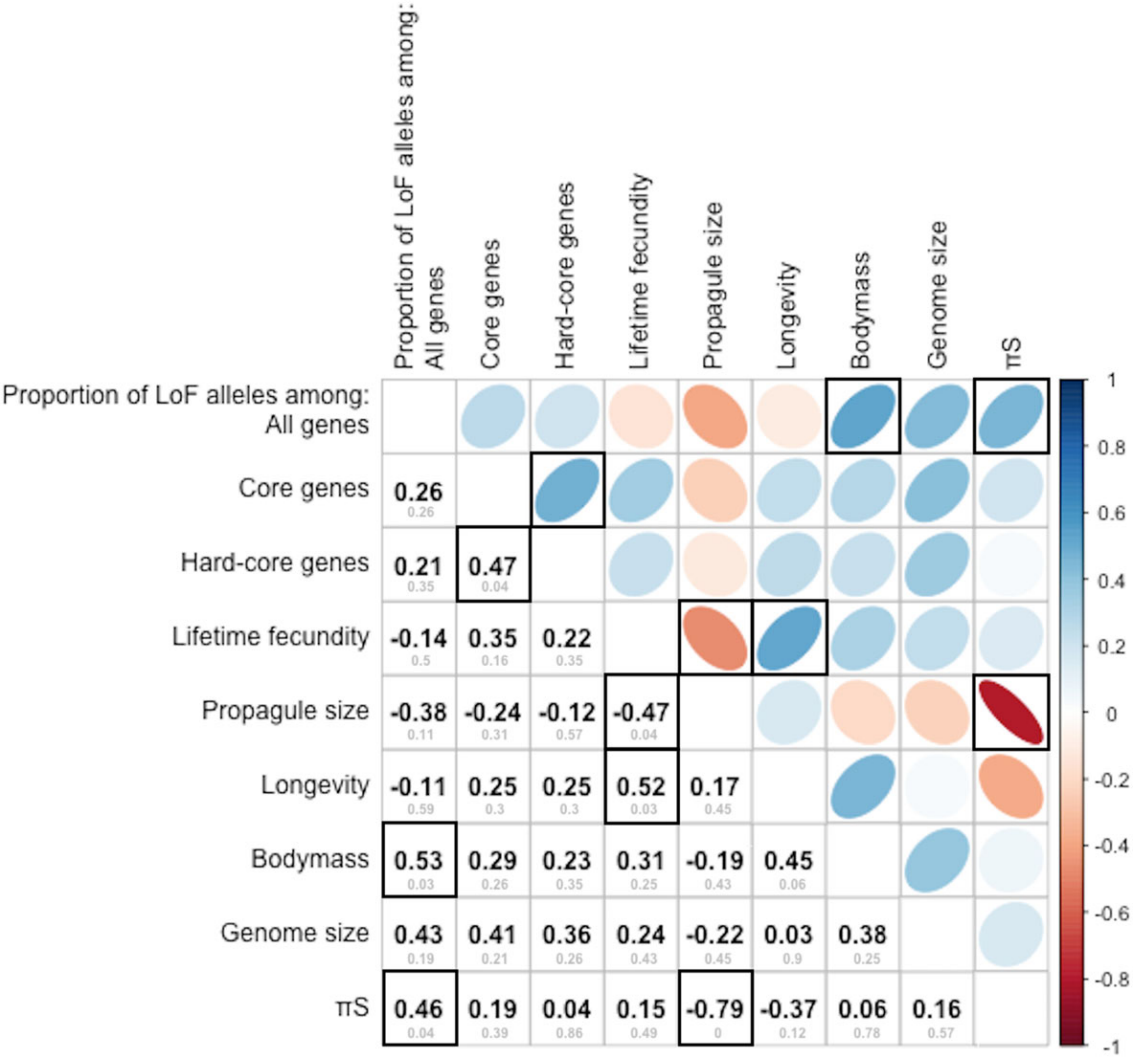
Correlations between mean proportions of LoF alleles among all, core, and hard-core genes and life-history traits. Blue indicates a positive relationship, red indicates a negative relationship, and color intensity is proportional to Spearman’s correlation coefficients, which are also presented below the diagonal together with *p*-values (in grey), corrected for multiple testing using BH procedure. Correlations that are significant (α < 0.05) are framed

We related the mean proportion of LoF alleles in a species to its lifetime fecundity (Fig. 1) and to other life-history traits, as well as to genome size and *π*_*S*_ (Fig. 2). For hard-core genes, this proportion shows no significant correlation with any of the traits. No correlations were also observed when different types of LoF alleles were considered separately (Additional file 6: Figure S1 and Additional file 7: Figure S2) for hard-core genes.

We also performed the analysis of the relationship between the proportion of LoF alleles in hard-core genes and species lifetime fecundity with two additional restrictions. First, a stricter quality threshold was imposed (at least 20X coverage). Second, only the last 100 nucleotides of each gene were taken into account, as a proxy for last exon, where NMD does not act [13]. These restrictions did not affect the key pattern that we observed (Additional file 8: Figure S3 and Additional file 9: Figure S4).

## Discussion

We investigated the strength of negative selection across a wide variety of metazoan species. This strength was assayed through the prevalence of LoF alleles of essential genes in genotypes of individuals. Frequencies of such alleles are generally quite low, and the data on recessive lethals in *Drosophila* populations suggest that coefficients of selection against them, in the heterozygous state, are ~ 1% [14]. Thus, the frequencies of such alleles are likely to be close to the deterministic mutation-selection equilibrium even in the smallest natural populations [15], which almost always have *N*_*e*_ > = 10^4^. In other words, the prevalence of such LoF alleles should be essentially independent of the effective sizes of natural populations. Indeed, in great apes the prevalence of LoF alleles does not depend on the effective population size [16]. Our analysis also found no correlation between the proportion of genes carrying LoF mutations and π_S_, an estimator of the effective population size. From this perspective, LoF alleles of important genes are radically different from missense alleles of all protein-coding genes, which are more prevalent in species with low effective population sizes due to inefficient selection against slightly deleterious mutations [17].

The mean proportion of LoF alleles of all genes across all species was 2.21%, which is consistent with the figures for primates, from ~ 0.7% in *Homo sapiens* to ~ 2.2% in *Pongo abelii* [16]. The proportion of frameshift indels exceeded the proportion of nonsense substitutions by a factor of ~ 4.9, which is consistent with the range of values obtained in [16], 1.7–4.7*.*

We observed no strong correlations between the prevalence of LoF alleles in all, core, or hard-core genes and lifetime fecundity or any other life-history trait of a species. This suggests that random mortality in highly prolific species may negate a higher opportunity for natural selection in them.

Of course, the prevalence of LoF variants must be proportional to the mutation rate. Could this fact mask the positive dependence of the strength of negative selection on the lifetime fecundity? This seems to be unlikely. Indeed, in order to explain our result in this way, one needs to assume that high-fecundity species have higher mutation rates. However, no data support this hypothesis. In fact, there are weak correlations of the opposite sign, as high-fecundity species tend to have higher *N*_*e*_ [3], and species with higher *N*_*e*_ tend to have lower mutation rates [18]. We also observe no strong correlation between the prevalence of LoFs and π_S_, which must depend linearly on the mutation rate.

Our analysis should not be confounded with the studies of the impact of random drift on the action of weak selection with |s| ~ 1/*N*_*e*_ [19]. The efficiency of weak negative selection declines in small populations, where more polymorphisms become effectively neutral [17, 20]. In contrast, negative selection against the majority of even heterozygous LoF variants is sufficiently strong [14, 15] to make their dynamics essentially independent of the random drift even in the smallest natural populations ([16] and Fig. 2).

## Conclusions

Our results suggest that a heterozygous LoF variant within a particular gene causes the same relative reduction of fitness in species with drastically different lifetime fecundities and opportunities for selection. This invariance is puzzling. Could it be a consequence of the evolutionary optimization of fecundity and other LHTs? If all species possess the values of LHTs that lead to the highest fitness, given their particular constraints, this may lead to the strongest possible negative selection. Still, it is not clear why the strongest possible selection turns out to be equally strong in cods and elephants.

## Reviewers’ comments

### Reviewer’s report 1: Nicolas Galtier, Université de Montpellier, Montpellier

Reviewer’s comments: This interesting article analyses the prevalence of loss­of­function alleles in the transcriptome of hundreds of individuals from 32 diverse species of animals. No relationship between prevalence of LOF mutations and fecundity is found, which is inconsistent with the prediction of more efficient selection in high­fecundity species. The manuscript discusses possible biological explanations to these unexpected results ­ e.g. high random mortality in high­fecundity species. Below I suggest checking a bit more deeply a couple of methodological issues, and the assumption of mutation/selection equilibrium.

Author’s response: *We thank the reviewer for the comments that allowed us to improve the manuscript.*

1. Transcriptomic data. LOF mutations in this analysis were identided based on a population transcriptomic data set. This is arguably suboptimal. First, mRNAs can differ from DNA due to transcription/splicing noise, which might be non­random ­ e.g., in case of misleading/ambiguous splicing signals. This might result in spurious LOF allele calls. The 5X threshold that was used is not a particularly stringent one; variants supported by as few as one or two reads could well be validated here. Correctly calling indels is typically more difficult than SNPs. I would strongly suggest analyzing and controlling for the effect of sequencing coverage on LOF allele call rate.
  Author’s response: *We absolutely agree that transcriptomic data are not optimal for studying LoF alleles. Unfortunately, there’s no consistent genomic data on populations of a large enough number of species. Thus, we decided to address the problem using the available data, with a thought in mind to revisit the results when enough genomic data become available. We tried a more strict coverage threshold, which did not affect our results (see the revised text).*
2. Secondly, it should be noted that mRNAs carrying a nonsense mutation are normally degraded by the nonsense mediated decay (NMD) pathway. NMD is documented in humans and yeast and is presumably ancestral to animals. Whether it is equally effective in all the species analyzed here is uncertain. Also, NMD in humans does not affect the 3′­ most exon, or single exon genes, so the effect might be dependent on gene structure and exon number/length distribution. It could be useful to check the pattern of LOF mutation distribution across coding sequence length, and how this might be related with NMD.
  Author’s response: *We agree that NMD decay may affect our results. Due to the lack of genomic sequences, it is hard to determine exonic structure of genes. Thus, we performed a somehow inaccurate test for robustness of our results by focusing on the last 100 nucleotides of genes as a proxy for the last exon (see the revised text).*
3. The mutation/selection equilibrium hypothesis. It is stated that the prevalence of LOF mutations is probably independent on effective population size (*N*_*e*_) because the associated selective effect, which would be of the order of 0.01, is much higher than the inverse of *N*_*e*_ in nearly all species. However, at mutation/selection equilibrium, the expected frequency of deleterious alleles is *q = u/s*, where *u* is the mutation rate and *s* the selection coefficient. *q* is here found to be of the order of 0.01, so assuming *s* = 0.01, we get *u* ~ 10^− 4^ per gene, i.e., ~ 10^− 7^ per base pair. This is order of magnitude higher than documented point mutation and insertion/deletion rates in animals (e.g. Sung et al. 2016 G3 6: 2583–2591). There seems to be a contradiction here, unless I’m mistaken. If, however, the selection coefficient was variable, some LOF mutations being only slightly deleterious, then these would be disproportionately abundant in the set of observable mutations. In this case one would predict an effect of *N*_*e*_ on the frequency of LOF mutants. The results of this analysis are again inconsistent with this prediction (Fig. 2), whereas a strong and significant relationship has been detected between π_N_/π_S_ and proxies for *N*_*e*_ with this data set (Romiguier et al. 2014). So, I don’t know what to think.
  Author’s response: *Estimate of s ~ 0.01 is based on the data on recessive lethals in Drosophila melanogaster*. *For hard-core genes in our analysis, q was found to be 0.0022, which implies a mutation rate of 2 × 10*^*− 8*^ *per nucleotide, which is high, but not as high as the reviewer assumes. Note, that this estimate is rather imprecise, and the real mutation rate can easily be several times lower (or higher). We apologize for a typo in the Y-axis label in* Fig. 1 *(noticed by the second reviewer), where ‘%’ was not needed.*
4. The analysis calls both heterozygous and homozygous LOF genotypes, but the manuscript does not present the detailed results. According to the hypothesis of strong selection, and knowing that LOF mutations are usually assumed to be highly recessive (especially in core genes), one would expect a strong departure from Hardy­Weinberg, i.e., zero or nearly zero homozygous LOF.
  Author’s response: *Our analysis is based on “hard-core” genes which, by our definition, exclude genes at which homozygous LoF alleles were observed in at least one species. Thus, we cannot study deviations from the HW expectations for this set. We agree with the reviewer that to compare homo- and heterozygous effects of LoF alleles would be very interesting, however, this analysis would require a much large number of genotypes per species.*
5. Haplodiploids Hymenoptera have been removed from the data set because males in these species are haploids, so that selection against recessive mutations is expected to be stronger than in other species. It would be interesting to analyses these species, though, precisely because we have the prediction that LOF mutations should be very rare (is that the case?). Actually, one could think of optimizing the pipeline based on the criterion of having much less LOF mutants in hymenoptera than in other species. (By the way: the text says that termites are haplodiploids but this is not true. Termites could safely be included in the set of regular species).
  Author’s response: *Sorry, we now include termites into analysis.*

### Reviewer’s report 2: I. King Jordan, Georgia Institute of Technology, Atlanta

Reviewer’s comments: Bezmenova and colleagues report on the relationship between the strength of selection, as measured by the proportion of loss of function (LoF) alleles, and average lifetime fecundity in 32 metazoan species. Contrary to predictions from population genetic theory, they find no correlation between the strength of selection and fecundity. Possible reasons for this unexpected result are explored. The work appears to be technically sound (but see several of the specific questions in the Minor issues section regarding the need for some clarifications). The main finding represents a quite interesting, if difficult to explain, contribution to the emerging discipline of population genomics. Overall, work of this kind is important, and publication of a negative result, such as reported here, should serve to stimulate further research in this area. For those reasons, I am support publication of the manuscript in Biology Direct. Below, I provide comments intended to amplify the discussion of the results along with questions and suggestions regarding the presentation of the data.

Author’s response: *We thank the reviewer for the comments that allowed us to improve the manuscript.*

1. The manuscript makes use of a large data set of transcriptomes that was previously reported in a 2014 paper on the population genomics and genetic diversity of animals (Rominguier et al. Nature 515: 261). Presumably, the LoF variant versus fecundity approach taken in this manuscript addresses a question that was left open by the previous report. If so, it would be helpful to state this explicitly in the manuscript and to point out how the results of the new analysis compare to, or add to, the findings from the previous study.
  Author’s response: *Indeed, Rominguier* et al. *produced and studied this data set for a very different purpose. They were interested in the relationships of the level of genetic diversity within a species, determined primarily be the effective population size and the mutation rate, with different life-history traits. In contrast, we investigate the efficacy of strong negative selection and its dependence on the species life-time fecundity.*
2. The manuscript would benefit from a comparison of LoF variants with mutation rate. This issue is treated in the Discussion, but no data on mutation rate are presented. Instead, the related features of *N*_*e*_ and π_S_ are discussed. I suspect that such data should be available for many of the species analyzed in the manuscript.
  Author’s response: *Unfortunately, the species sequenced in Rominguier* et al. *(Nature, 2014) are all non-model, so that no data on mutation rates are unavailable for almost all of them.*
3. The notion of higher random mortality, and lower paternal investment, in high­fecundity species cited by the authors as an explanation for their results is reminiscent of the ecological concept of r/K selection theory. An articulation of the similarities and differences of the authors own argument with this widely known concept could be illuminating.
  Author’s response: *The r/K selection paradigm is no longer widely accepted by ecologists (Reznik* et al. *Ecology, 2002*)*.*
4. It seems that the interpretation provided for the results depends on an additive model of selection with LoF heterozygotes half as fit as wild­type homozygotes. Is this in fact the case? Can dominance effects, where LoF heterozygotes are less visible to selection, partially explain these results?
  Author’s response: *Our analysis is based only on those genes for which LoF homozygotes were not observed in all the species studied. We used this as a proxy to gene essentiality. To study the degree of recessivity of LoF alleles would be very interesting; however, it requires a much large number of samples of genotypes from every species.*
5. The statement in the Results that “No correlations were also observed when different types of LoF variants were considered separately”, presumably meaning no significant correlations, is contradicted by the results shown in Additional file 6: Figure S1, where propagule size and π_S_ appear to be significantly correlated with the proportion of nonsense alleles among all genes.
  Author’s response: *Thank you! We have corrected the wording and stated that no correlations were observed when different types of LoF variants were considered separately for hard-core genes, which was originally the point.*
6. The paper concludes with a tentative assertion regarding the role of random mortality in mitigating opportunities for the action of natural selection in high­fecundity species. However, no direct support is provided for this and the authors are understandably measured in presenting the argument. I can’t help but wonder if there is a missed opportunity here for the use of population genetic modeling, both to establish the null expectation and to explore the possible effects of different forces on the LoF versus fecundity relationship. While I suspect this would not be too difficult given the authors’ expertise, it is intended as an optional suggestion and left to the authors’ discretion to consider.
  Author’s response: *To perform this analysis, one needs to know the variance of the expected fitness (given genotype) in the population, and to compare it with the variance of fitness of individuals (zero variance of expected fitnesses of genotypes would indicate the absence of natural selection, and equality of the two variances would indicate that random mortality is absent). Unfortunately, this is impossible. This would be very, indeed, interesting, but, unfortunately, the necessary data are not available.*
7. Another optional suggestion relates to the brevity of the Results section and the large amount of data relegated to the Supplement. Given the lack of space constraints in Biology Direct, the authors may consider including more of the relevant results, and discussion of them, in the main body of the manuscript, particularly Additional file 6: Figure S1 and Additional file 7: Figure S2.
  Author’s response: *The amount of data available was not enough to attribute much importance to the differences between LoF alleles shown in* Additional file 6*: Figure S1 and* Additional file 7*: Figure S2. By contrast, we hope that our key result is meaningful. That is why we would rather not include this figures into the main text.*

Minor issues:

1. The introduction states “...to ensure a constant long-term population size, pre­reproductive mortality in a species must be inversely proportional to its average lifetime fecundity.” Why inversely proportional? Shouldn’t it be just proportional, i.e. more pre­reproductive mortality in higher­fecundity species?
2. The expectation of a correlation between the strength of selection, as measured by the proportion of LoF alleles, and average lifetime fecundity is made clear. It would also help to explicitly state the expected direction of the correlation between the LoF test statistic and fecundity. Presumably it should be negative with higher­fecundity species having proportionally fewer LoF alleles.
3. I was confused by the use of alleles (versus genes or loci) in the explanation of the formula for the proportion of LoF alleles. If NLoFHom is the number of alleles with a homozygous variant, i.e. 2 alleles per gene, then why do you need 2NLoFHom in the formula instead of just NLoFHom.
4. It is not clear why the authors used both de novo assembly of transcripts and mapping to reference genomes, which seems to be the approach used to call LoF variants. Was de novo assembly just used to define the core gene sets?
  Author’s response: *we mapped reads to reference transcriptome assemblies, as genomes are unavailable for studied species.*
5. The minimum values for each of the three classes of LoF variants shown in Table 1 is more than an order of magnitude higher than the minimum value shown for the three classes. Is this a typo?
6. It is not clear why the numbers of species analyzed for all genes and hard­core genes differs in Fig. 1.
  Author’s response: *some dots on the chart overlap; we tried to fix this issue by making dots transparent.*
7. The legends of Figs. 1 and 2 refer to Spearman’s correlation coefficients, which is typically denoted as the Greek letter rho (ρ) or r_s_. But Fig. 1 shows the symbol R2, which is typically used for the coefficient of determination. This can lead to confusion in interpreting the significance of the results.
8. Related to the previous comment, evidence for the lack of statistical significance for the correlation between LoF variants fecundity and reported by the authors is not made explicitly clear in the manuscript. It would be helpful if P­values are shown and if the statistical approaches used are described in the Methods section.
  Author’s response: *we thank the reviewer for pointing out these minor issues; we have fixed them.*

## Additional files

Additional file 1: **Table S1.** Sequence Read Archive IDs of studied transcriptomes. (XLSX 53 kb)

Additional file 2: **Table S2.** Transcriptomes assembly and annotation statistics. (XLSX 12 kb)

Additional file 3: **Table S3.** Life-history traits, genome sizes and polymorphism of species, where available. (XLSX 11 kb)

Additional file 4: **Table S4.** Proportions of LoF alleles among all, core and hard-core genes for each individual. (XLSX 20 kb)

Additional file 5: **Table S5.** Mean proportions of LoF alleles among all, core and hard-core genes for each species. (XLSX 12 kb)

Additional file 6: **Figure S1.** Correlations between mean proportions of nonsense alleles among all, core, and hard-core genes and life-history traits. Blue indicates a positive relationship, and red, a negative relationship; color intensity is proportional to Spearman’s correlation coefficients, which are also presented below the diagonal together with *p*-values (in grey), corrected for multiple testing using BH procedure. Correlations that are significant (α < 0.05) are framed. (PDF 871 kb)

Additional file 7: **Figure S2.** Correlations between mean proportions of frameshift alleles among all, core, and hard-core genes and life-history traits. Blue indicates a positive relationship, and red, a negative relationship; color intensity is proportional to Spearman’s correlation coefficients, which are also presented below the diagonal together with p-values (in grey), corrected for multiple testing using BH procedure. Correlations that are significant (α < 0.05) are framed. (PDF 884 kb)

Additional file 8: **Figure S3.** The mean proportions of LoF alleles against lifetime fecundity in all (green) and in hard-core (orange) genes for each species with 20X coverage threshold (Spearman’s correlation coefficients are − 0.04 and 0.26, respectively; p-values are 0.81 and 0.14). (PDF 285 kb)

Additional file 9: **Figure S4.** The mean proportions of LoF alleles in the last 100 nucleotides of each gene against lifetime fecundity in all (green) and in hard-core (orange) genes for each species (Spearman’s correlation coefficients are − 0.19 and − 0.05, respectively; p-values are 0.28 and 0.76). (PDF 268 kb)
