# Supplementary material for: Prevalence of loss-of-function alleles does not correlate with lifetime fecundity and other life-history traits in metazoans
Source: Biol Direct. 2018 Mar 2;13:4. doi: 10.1186/s13062-018-0206-9 (PMC5834895; doi:10.1186/s13062-018-0206-9)
Supplement: Supplementary file 6 — Figure S1. Correlations between mean proportions of nonsense alleles among all, core, and hard-core genes and life-history traits. Blue indicates a positive relationship, and red, a negative relationship; color intensity is proportional to Spearman’s correlation coefficients, which are also presented below the diagonal together with p-values (in grey), corrected for multiple testing using BH procedure. Correlations that are significant (α < 0.05) are framed. (PDF 871 kb) [file 13062_2018_206_MOESM6_ESM.pdf]

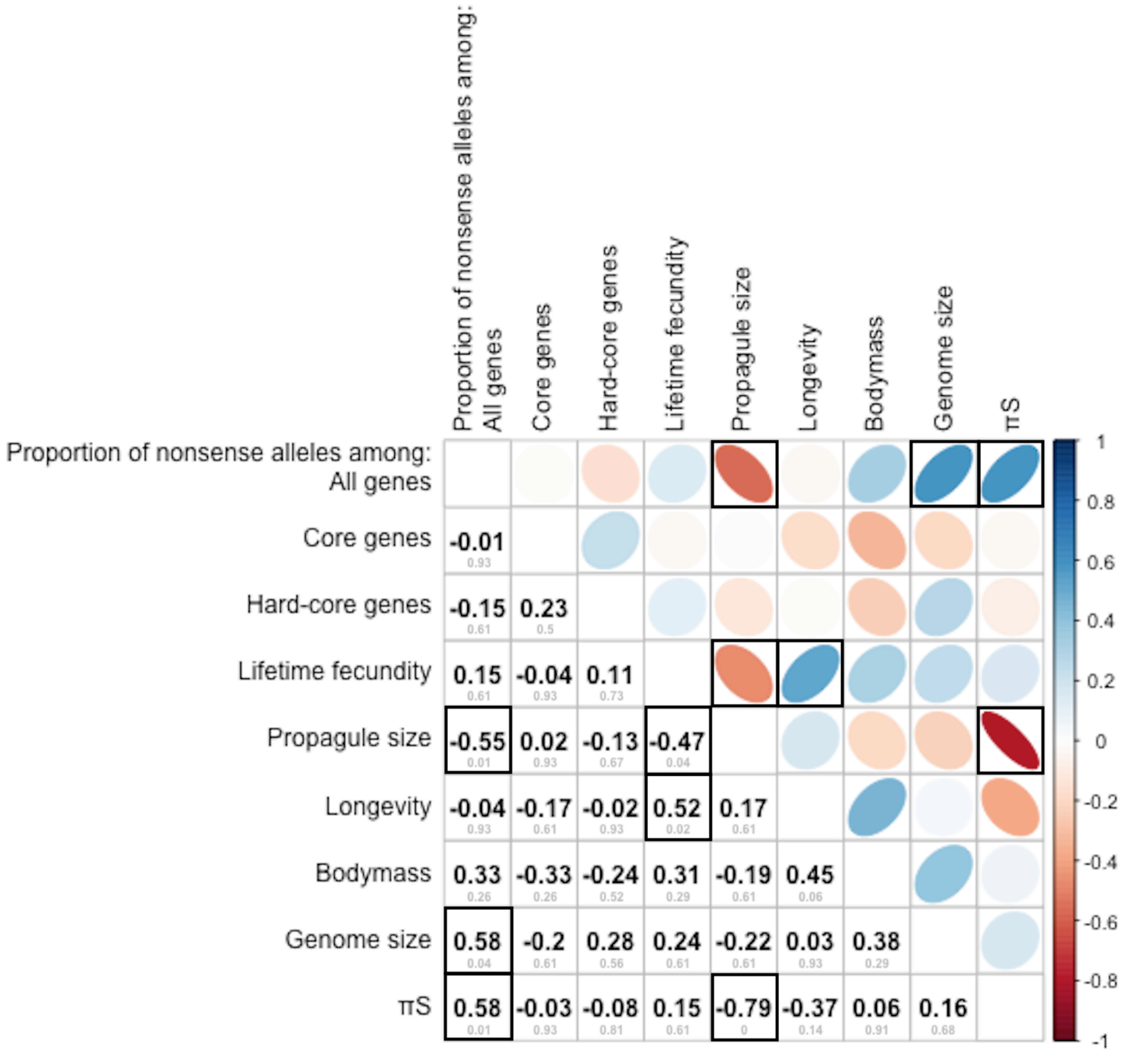

Figure S1. Correlations between mean proportions of nonsense alleles among all, core, and hard-core genes and life-history traits. Blue indicates a positive relationship, and red, a negative relationship; color intensity is proportional to Spearman's correlation coefficients, which are also presented below the diagonal together with p-values (in grey), corrected for multiple testing using BH procedure. Correlations that are significant ($\alpha < 0.05$) are framed.
