# Supplementary material for: Prevalence of loss-of-function alleles does not correlate with lifetime fecundity and other life-history traits in metazoans
Source: Biol Direct. 2018 Mar 2;13:4. doi: 10.1186/s13062-018-0206-9 (PMC5834895; doi:10.1186/s13062-018-0206-9)
Supplement: Supplementary file 9 — Figure S4. The mean proportions of LoF alleles in the last 100 nucleotides of each gene against lifetime fecundity in all (green) and in hard-core (orange) genes for each species (Spearman’s correlation coefficients are − 0.19 and − 0.05, respectively; p-values are 0.28 and 0.76). (PDF 268 kb) [file 13062_2018_206_MOESM9_ESM.pdf]

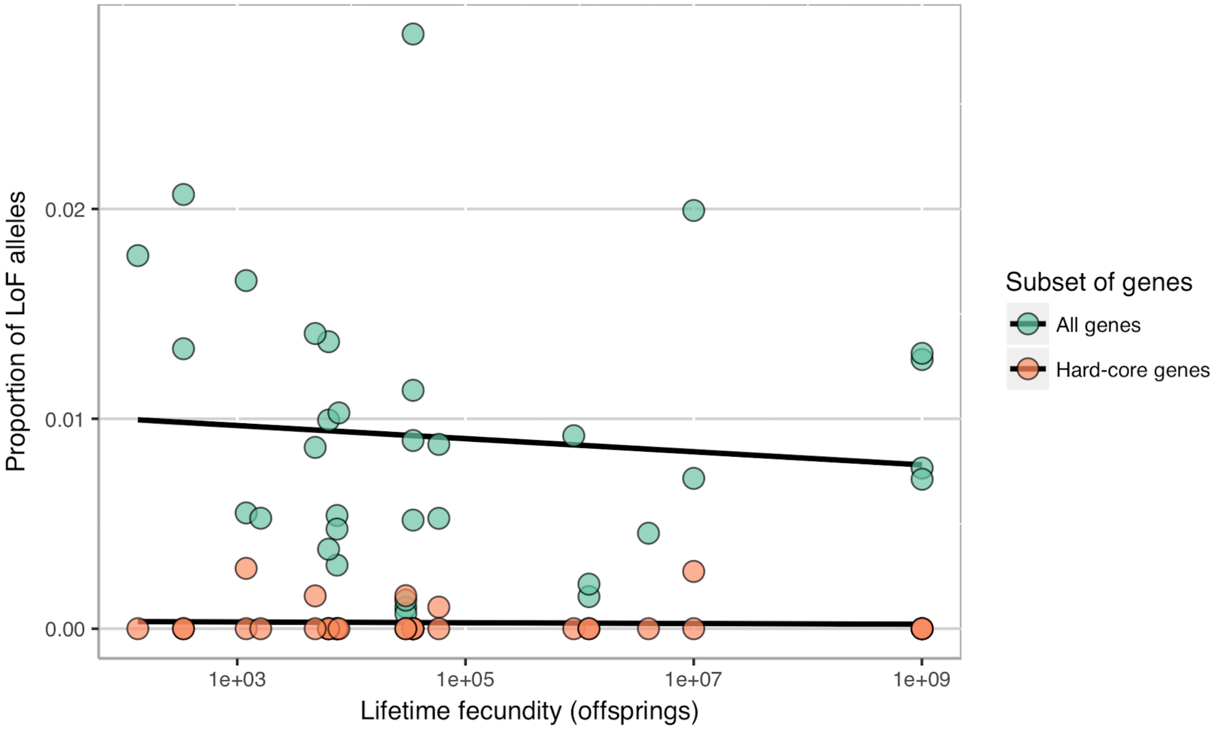

Figure S4. The mean proportions of LoF alleles in the last 100 nucleotides of each gene against lifetime fecundity in all (green) and in hard-core (orange) genes for each species (Spearman's correlation coefficients are -0.19 and -0.05, respectively; p-values are 0.28 and 0.76).
