# Supplementary material for: Prevalence of loss-of-function alleles does not correlate with lifetime fecundity and other life-history traits in metazoans
Source: Biol Direct. 2018 Mar 2;13:4. doi: 10.1186/s13062-018-0206-9 (PMC5834895; doi:10.1186/s13062-018-0206-9)
Supplement: Supplementary file 8 — Figure S3. The mean proportions of LoF alleles against lifetime fecundity in all (green) and in hard-core (orange) genes for each species with 20X coverage threshold (Spearman’s correlation coefficients are − 0.04 and 0.26, respectively; p-values are 0.81 and 0.14). (PDF 285 kb) [file 13062_2018_206_MOESM8_ESM.pdf]

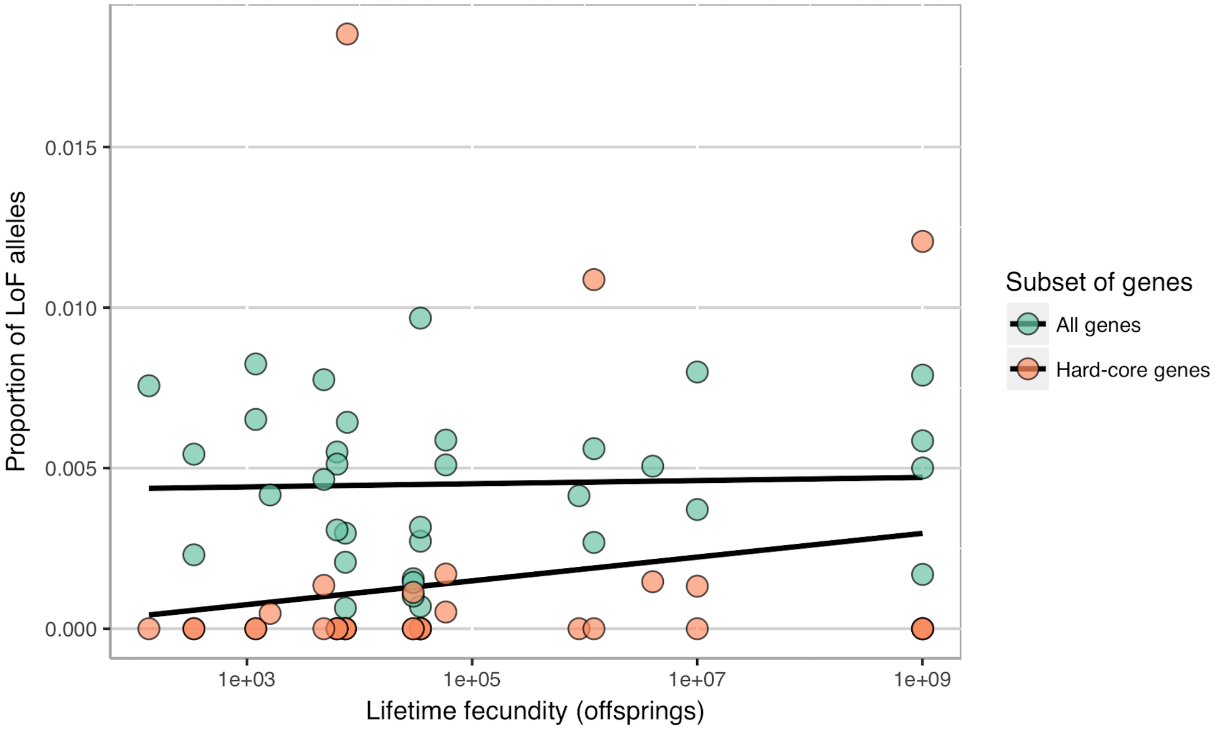


**Figure S3.** The mean proportions of LoF alleles against lifetime fecundity in all (green) and in hard-core (orange) genes for each species with 20X coverage threshold (Spearman's correlation coefficients are -0.04 and 0.26, respectively; p-values are 0.81 and 0.14).
